# Impact of misspecifying the distribution of a prognostic factor on power and sample size for testing treatment interactions in clinical trials

**DOI:** 10.1186/1471-2288-13-21

**Published:** 2013-02-15

**Authors:** William M Reichmann, Michael P LaValley, David R Gagnon, Elena Losina

**Affiliations:** 1 Department of Orthopedic Surgery, Brigham and Women’s Hospital, Boston, MA, USA; 2 Department of Biostatistics, Boston University School of Public Health, 801 Massachusetts Avenue, 3rd Floor, Boston, MA, USA; 3 Massachusetts Veterans Epidemiology Research and Information Center, VA Cooperative Studies Program, Boston, MA, USA; 4 Orthopedic and Arthritis Center for Outcomes Research, Brigham and Women’s Hospital, 75 Francis Street, BC 4-4016, Boston, MA, 02115, USA

**Keywords:** Simulation design, Interaction, Conditional power, Adaptive design, Sample size re-estimation

## Abstract

**Background:**

Interaction in clinical trials presents challenges for design and appropriate sample size estimation. Here we considered interaction between treatment assignment and a dichotomous prognostic factor with a continuous outcome. Our objectives were to describe differences in power and sample size requirements across alternative distributions of a prognostic factor and magnitudes of the interaction effect, describe the effect of misspecification of the distribution of the prognostic factor on the power to detect an interaction effect, and discuss and compare three methods of handling the misspecification of the prognostic factor distribution.

**Methods:**

We examined the impact of the distribution of the dichotomous prognostic factor on power and sample size for the interaction effect using traditional one-stage sample size calculation. We varied the magnitude of the interaction effect, the distribution of the prognostic factor, and the magnitude and direction of the misspecification of the distribution of the prognostic factor. We compared quota sampling, modified quota sampling, and sample size re-estimation using conditional power as three strategies for ensuring adequate power and type I error in the presence of a misspecification of the prognostic factor distribution.

**Results:**

The sample size required to detect an interaction effect with 80% power increases as the distribution of the prognostic factor becomes less balanced. Misspecification such that the actual distribution of the prognostic factor was more skewed than planned led to a decrease in power with the greatest loss in power seen as the distribution of the prognostic factor became less balanced. Quota sampling was able to maintain the empirical power at 80% and the empirical type I error at 5%. The performance of the modified quota sampling procedure was related to the percentage of trials switching the quota sampling scheme. Sample size re-estimation using conditional power was able to improve the empirical power under negative misspecifications (i.e. skewed distributions) but it was not able to reach the target of 80% in all situations.

**Conclusions:**

Misspecifying the distribution of a dichotomous prognostic factor can greatly impact power to detect an interaction effect. Modified quota sampling and sample size re-estimation using conditional power improve the power when the distribution of the prognostic factor is misspecified. Quota sampling is simple and can prevent misspecification of the prognostic factor, while maintaining power and type I error.

## Background

Randomized controlled trials (RCTs) are the gold standard for evaluating the efficacy of a treatment or regimen. While for most RCTs the primary hypothesis is the overall comparison of two (or more) treatments, there has been a continuing discussion over the last two decades about the use of subgroup analyses and formal tests of interaction in RCTs [1-7]. According to the most recent CONSORT statement, which was published in 2010, the analysis of subgroups should be pre-planned and accompanied by a formal test of interaction [8]. However, systematic reviews of medical and surgical RCTs have shown that many of the analyses of subgroups in RCTs have not been pre-planned and have not been accompanied by a formal test of interaction [1-4]. The percentage of trials reporting their results using a formal interaction test was 13% in 1985 [1], 43% in 1997 [2], 6% from 2000 to 2003 [3], and 27% from 2005 to 2006 [4].

Investigators planning subgroup analyses within the framework of RCTs are encouraged to design RCTs to detect interaction effects using a formal interaction test. While statistical software such as nQuery Advisor and SAS can handle power and sample size calculations for detecting interaction effects, a modest body of literature exists describing the effect of the magnitude of the interaction effect and distribution of the prognostic factor have on power and sample size. Two articles by Brookes and colleagues showed that there is low power to detect an interaction, scaled as a contrast of cell means, when a study is powered only to detect the main effect unless size of the interaction effect is nearly twice as large as the main effect [6,7]. They also showed that power for the interaction test is maximized when the prognostic factor is distributed evenly.

There are many instances in which investigators may be interested in studying the interaction between a prognostic factor and treatment. For example, an investigator is interested in studying the effect in improving functional limitation in persons with meniscal tear and concomitant knee osteoarthritis (OA). For this disease, the two treatment choices are performing arthroscopic partial meniscectomy (APM) and physical therapy (PT). However, the investigator also hypothesizes that the effect of APM compared to PT on functional limitation varies by knee OA severity. In this case, knee OA severity is the prognostic factor and how it is distributed will impact the sample size required to detect the interaction between treatment and OA severity.

This article has three objectives. First, we sought to describe differences in power and sample size requirements across alternative distributions of a prognostic factor and magnitudes of the interaction effect. Second, we describe the effect of misspecification of the prognostic factor distribution and how such misspecification affects the power to detect an interaction effect. Third, we describe and discuss three methods of handling misspecification of the prognostic factor distribution by potential readjustment of the sample size or strategy during a trial. Two of these methods are sampling-based and do not require an interim statistical testing of the outcome. The third method uses a two-stage adaptive design approach that re-estimates the sample size based on the conditional power at an interim analysis where 50% of the patients have been enrolled.

## Methods

### Overview

We conducted an analysis examining the impact of how different distributions of a dichotomous prognostic factor affect the power (and sample size needed to obtain 80% power) to detect an interaction between the prognostic factor and treatment in RCTs. We also studied the impact of misspecifying (positive and negative misspecifications) the distribution of the prognostic factor on power and sample size. We varied the magnitude of the interaction effect, the distribution of the prognostic factor, and the magnitude of the misspecification. Lastly, we compare three methods for ensuring appropriate overall power and type I error under the misspecification of the distribution of the prognostic factor. These methods are quota sampling, modified quota sampling, and sample size re-estimation using conditional power.

### Specification of key parameters used in the paper

#### Treatment variable

The treatment variable was distributed as a binomial variable (active vs. placebo) with probability of 0.5. For the purposes of this paper the treatment variable was assumed to always have a balanced distribution (i.e. 50% on level 1 receiving active treatment; 50% on level 2 receiving placebo). For illustration purposes, we assumed that APM was the active treatment and that PT was the placebo.

#### Prognostic factor

The prognostic factor was defined as dichotomous variable with *k*_*j*_ representing the *j*^*th*^ level of the prognostic factor. When referring to the distribution of the prognostic factor we indicated the percentage in the *k*_*1*_ level of the prognostic factor, defined as *p*_*1*_. We varied *p*_*1*_ from 10% to 50% in 10% increments.

#### Misspecification of the prognostic factor

The misspecification of the prognostic factor was defined by the parameter *q*. The misspecification could be positive or negative with negative misspecification implying less balance (more skew) and positive misspecification implying more balance (less skew). For example, if the planned distribution of the prognostic factor was 20% and the actual distribution of the prognostic factor was 25%, then the misspecification of the prognostic factor (*q*) was +5%. Possible values of *q* were −15%, -5%, 0 (i.e. no misspecification), +5%, and +15%.

#### Outcome variable

We assumed that our outcome variable was continuous and normally distributed. In our example, the outcome can be interpreted as the improvement in function after APM or PT as measured by a score or scale. We specified the mean improvement for all four possible combinations of treatment and the prognostic factor. We considered two different values (25 and 15) for the mean improvement in the active/*k*_*1*_ treatment/prognostic factor combination (i.e. APM/mild knee OA severity). The mean improvement in the active/*k*_*2*_, placebo/*k*_*1*_, and placebo/*k*_*2*_ groups were held constant at 5, 5, and 0 respectively. We assumed a common standard deviation (*σ*) of 10 for all four combinations.

#### Magnitude of the interaction

We defined the magnitude of the interaction between prognostic factor and treatment effect according to the method by Brookes and colleagues [6,7]. Let *μ*_*ij*_ be mean improvement in the *i*^*th*^ treatment and *j*^*th*^ level of the prognostic factor. We then defined the treatment efficacy in the *j*^*th*^ level of the prognostic factor as:

(1) $$\delta_{j}=\mu_{1j}-\mu_{2j}$$

We then defined the interaction effect (denoted as *θ*) as follows:

(2) $$\theta =\delta_{1}-\delta_{2}=\left(\mu_{11}-\mu_{21}\right)-\left(\mu_{12}-\mu_{22}\right)$$

Thus *θ*, which served as the basis of our choice of mean improvement values, varied as *μ*_11_ varied. The magnitudes of the interaction effect that we considered were 15 and 5. The estimate of the interaction effect was defined as follows:

(3) $$\hat{\theta }=\left({\bar{x}}_{11}-{\bar{x}}_{21}\right)-\left({\bar{x}}_{12}-{\bar{x}}_{22}\right)$$

Then, the variance of the interaction effect under balanced treatment groups and distribution of the prognostic factor *p*_*1*_ can be derived as follows (note that *N* equals the total sample size for the trial):

(4) $$\begin{array}{l} VAR\left(\hat{\theta }\right)=VAR\left(\left({\bar{x}}_{11}-{\bar{x}}_{21}\right)-\left({\bar{x}}_{12}-{\bar{x}}_{22}\right)\right)\\ =VAR\left({\bar{x}}_{11}\right)+VAR\left({\bar{x}}_{21}\right)+VAR\left({\bar{x}}_{12}\right)+VAR\left({\bar{x}}_{22}\right)\\ =\frac{\sigma^{2}}{n_{11}}+\frac{\sigma^{2}}{n_{21}}+\frac{\sigma^{2}}{n_{12}}+\frac{\sigma^{2}}{n_{22}}\\ =\frac{\sigma^{2}}{0.5*p_{1}*N}+\frac{\sigma^{2}}{0.5*\left(1-p_{1}\right)*N}+\frac{\sigma^{2}}{0.5*p_{1}*N}\\ \;+\frac{\sigma^{2}}{0.5*\left(1-p_{1}\right)*N}\\ =\frac{4\sigma^{2}}{p_{1}*N}+\frac{4\sigma^{2}}{\left(1-p_{1}\right)*N}=\frac{4\sigma^{2}}{p_{1}*\left(1-p_{1}\right)*N} \end{array}$$

It is clear from the equation as the prevalence of the prognostic factor (*p*_*1*_) increases, the variance decreases, which would imply that the power increases for a fixed sample size.

### Initial sample size for interaction effects

The sample size required for the *i*^*th*^ treatment and *j*^*th*^ prognostic factor level to detect the interaction effect described under a balanced design (i.e. *p*_*1*_ = 0.5) with a two-sided significance level of *α* and power equal to 1– *β* has been previously published by Lachenbruch [9].

(5) nij=4σ2z1−β+z1−α22θ2

In these formulas *z*_*1–β*_ represents the *z*-value at the 1*–β* (theoretical power) quantile of the standard normal distribution and *z*_*1–α/2*_ represents the z-value at the 1*–*^*α*^*/*_*2*_ (probability of a type I error) quantile of the standard normal distribution. Under a balanced design with *p*_*1*_ = 0.5 we can just multiply *n*_*ij*_ by four to obtain the total sample size since there are four combinations of treatment and prognostic factor. A limitation of this formula is that it uses critical values from the standard normal distribution rather than the Student’s t-distribution as most statistical tests of interaction are performed using a t-distribution. To account for this we calculated the total sample size required to detect an interaction effect with a two-sided significance level of *α* and power equal to 1– *β*:

1. Use formula 5 (above) to calculate the sample size required for each combination of treatment and prognostic factor under a balanced design.

2. Calculate a new sample size $(n_{\mathrm{ij}}^{*})$ required for each combination of treatment and prognostic factor under a balanced design using the following formula 6 below. In this formula the z-critical values have been replaced with t-critical values with *n*_*ij*_ degrees of freedom.

(6) nij*=4σ2t1−β,nij−1+t1−α2,nij−12θ*2

3. Set *n*_*ij*_ equal to $n_{\mathrm{ij}}^{*}$ and repeat step 2.

4. Repeat step 3 until $n_{\mathrm{ij}}^{*}$ converges. This will usually occur after 2 or 3 iterations.

5. Lastly, to correct for imbalance in the prognostic factor, multiply $n_{\mathrm{ij}}^{*}$ by $\frac{1}{p_{1}\left(1-p_{1}\right)}$ to obtain the final total sample size *N*.

### Effect of misspecifying the distribution of the prognostic factor

The effect of misspecifying the distribution of the prognostic factor was evaluated using power curves. The formula used by Lachenbruch was extended to incorporate the Student’s t-distribution [9]. Power for the interaction test, where Ψ is the cumulative distribution function of the Student’s t-distribution, by actual prevalence of the prognostic factor (*p*_*1*_ + *q*) and magnitude of the interaction effect was calculated using equation 7 below.

(7) Power=1−ΨNp1+q1−p1−qθ24σ2−tα2,N−4

### Strategies for accounting for the misspecification of the distribution of the prognostic factor

#### Quota sampling

The quota sampling approach was performed using the following steps. First, for a given set of parameters, we would determine the sample size needed to detect an interaction effect with 80% power. We then fixed the number of participants to be recruited for each level of the prognostic factor. For example, if the final total sample size was 200 and the planned distribution of the prognostic factor was 30% in the *k*_*1*_ group and 70% in the *k*_*2*_ group then exactly 60 subjects would be recruited in the *k*_*1*_ group and 140 in the *k*_*2*_ group. This method removes the variability in the sampling distribution and ensures that the sampled prognostic factor distribution always matches what was planned for. Because of this approach, the observed distribution of the prognostic factor in the trial will always match the planned distribution and there will be no misspecification. However, this method may require turning away potential subjects because one level of the prognostic factor is already filled, delaying trial completion. Also, it may reduce the external validity of the overall treatment results as the trial subjects can become less representative of the unselected population of interest. Because of these limitations we also considered a modified quota sampling approach.

#### Modified quota sampling

The modified quota sampling approach was performed using the following steps. First, as in the quota sampling approach, the sample size needed to detect an interaction effect with 80% power was determined for the pre-specified parameters. Next, the simulated study enrolled the first ^*N*^*/*_*2*_ subjects. After the first ^*N*^*/*_*2*_ subjects were enrolled we tested to see if the sampling distribution of the prognostic factor was different from what was planned for using a one-sample test of the proportion. If this result was statistically significant at the 0.05 level then a quota sampling approach was undertaken for the second ^*N*^*/*_*2*_ subjects to be enrolled to ensure that the sampling distribution of the prognostic factor matched the planned distribution exactly. If the result was not statistically significant then the study continued to enroll normally, allowing for variability in the distribution of the prognostic factor.

#### Sample size re-estimation using conditional power

The last method for accounting for the misspecification of the distribution of the prognostic factor used the conditional power of the interaction test at an interim analysis to re-estimate the sample size. We modified the methods of Denne to carry out this procedure [10]. We assumed that the interim analysis occurred after the first ^*N*^*/*_*2*_ subjects were enrolle_*2*_) were determined by the O’Brien-Fleming alpha-spending function [11] using the SEQDESIGN procedure in the SAS statistical software package. We also used the SEQDESIGN procedure to calculate a futility boundary at the interim analysis (*b*_*1*_). Since these critical values are based on a standard normal distribution and not the student’s t-distribution we converted the critical values to those based on the student’s t-distribution. First, we converted the original critical values to the corresponding percentile of the standard normal distribution. We then converted these percentiles to the corresponding critical value of the Student’s t-distribution with *N-4* degrees of freedom.

At the interim analysis, if the absolute value of the interaction test statistic was less than the futility boundary (*t*_*1*_ < *b*_*1*_) then we stopped the trial for futility and considered the result of the trial to be not statistically significant. If the absolute value of the test statistic was greater than the interim critical value (*c*_*1*_) then we stopped the trial for efficacy and considered the result of the trial to be statistically significant. If absolute value of the test statistic was greater than *b*_*1*_ but less than *c*_*1*_ then we evaluated the conditional power and determined if sample size re-estimation was necessary. The following paragraphs outline this procedure.

The following is the conditional power formula proposed by Denne for the two group comparison of means:

(8) $$\mathrm{CP}=1-\Phi \left[\frac{c_{2}\sqrt{n_{2}}-z_{1}\sqrt{n_{1}}-\frac{\left(n_{t}-n_{1}\right)\delta }{\sigma }}{\sqrt{n_{t}-n_{1}}}\right]$$

Here, *c*_*2*_ is the final critical value, *n*_*2*_ is the sample size at the final analysis, *n*_*t*_ is the originally planned total sample size, *z*_*1*_ is the test statistic for the interaction at the interim analysis, *n*_*1*_ is sample size at the interim analysis, *δ* is the difference in means, and *σ* is the common standard deviation for the two groups. We updated the formula by replacing *z*_*1*_ with *t*_*1*_ (because the interaction test uses the Student’s t-distribution), *δ* (difference in means between groups) with *θ* (magnitude of the interaction effect), and Φ (cumulative distribution function of a standard normal distribution) with Ψ (cumulative distribution function of a student’s t-distribution). Recall that *p*_*1*_ is the proportion in the *k*_*1*_ group and *σ* is the common standard deviation:

(9) $$\mathrm{CP}=1-\Psi \left[\frac{c_{2}\sqrt{n_{2}}-t_{1}\sqrt{n_{1}}-\frac{\left(n_{t}-n_{1}\right)\theta \sqrt{p_{1}\left(1-p_{1}\right)}}{\sqrt{4\sigma^{2}}}}{\sqrt{n_{t}-n_{1}}}\right]$$

Initially *n*_*2*_ = *n*_*t*_ as conditional power is calculated as if you were to not re-estimate the sample size. The values of *θ*, *σ*, and *p*_*1*_ for the conditional power formula were estimated at the interim analysis. If the conditional power was less than 80% then a new *n*_*2*_ was estimated such that conditional power was 80% and a new final critical value, *c*_*2*_, was calculated as a function of the original final critical value, ${\tilde{c}}_{2}$, and the interim test statistic *t*_*1*_ using the following formula:

(10) $$c_{2}={\tilde{c}}_{2}\sqrt{\frac{\gamma_{2}-\gamma_{1}}{\gamma_{2}\left(1-\gamma_{1}\right)}}-t_{1}\sqrt{\left(\frac{\gamma_{1}}{\gamma_{2}}\right)}\left(\sqrt{\frac{\gamma_{2}-\gamma_{1}}{1-\gamma_{1}}}-1\right)$$

In equation 10, γ1=n1nt and γ2=n2nt so that the final critical value is also a function of *n*_*1*_, *n*_*2*_ (the new total sample size), and *n*_*t*_ (the original total sample size). Since all values except *n*_*2*_ are fixed, we can calculate the new critical value *c*_*2*_ for new final sample sizes *n*_*2*_. According to Denne, this method for re-estimating the sample size maintains the overall Type I error rate at *α* (equal to 0.05 in our case) [10]. The final sample size *n*_*2*_ and final critical value *c*_*2*_ were chosen so that the conditional power formula shown in equation 9 was equal to 80%. If the conditional power was greater than 80% at the interim analysis then we used the originally calculated *n*_*t*_ as the final sample size (*n*_*2*_ = *n*_*t*_) so that the final sample size was only altered to increase the conditional power to 80%.

### Validating the conditional power formula

**Figure 1:**
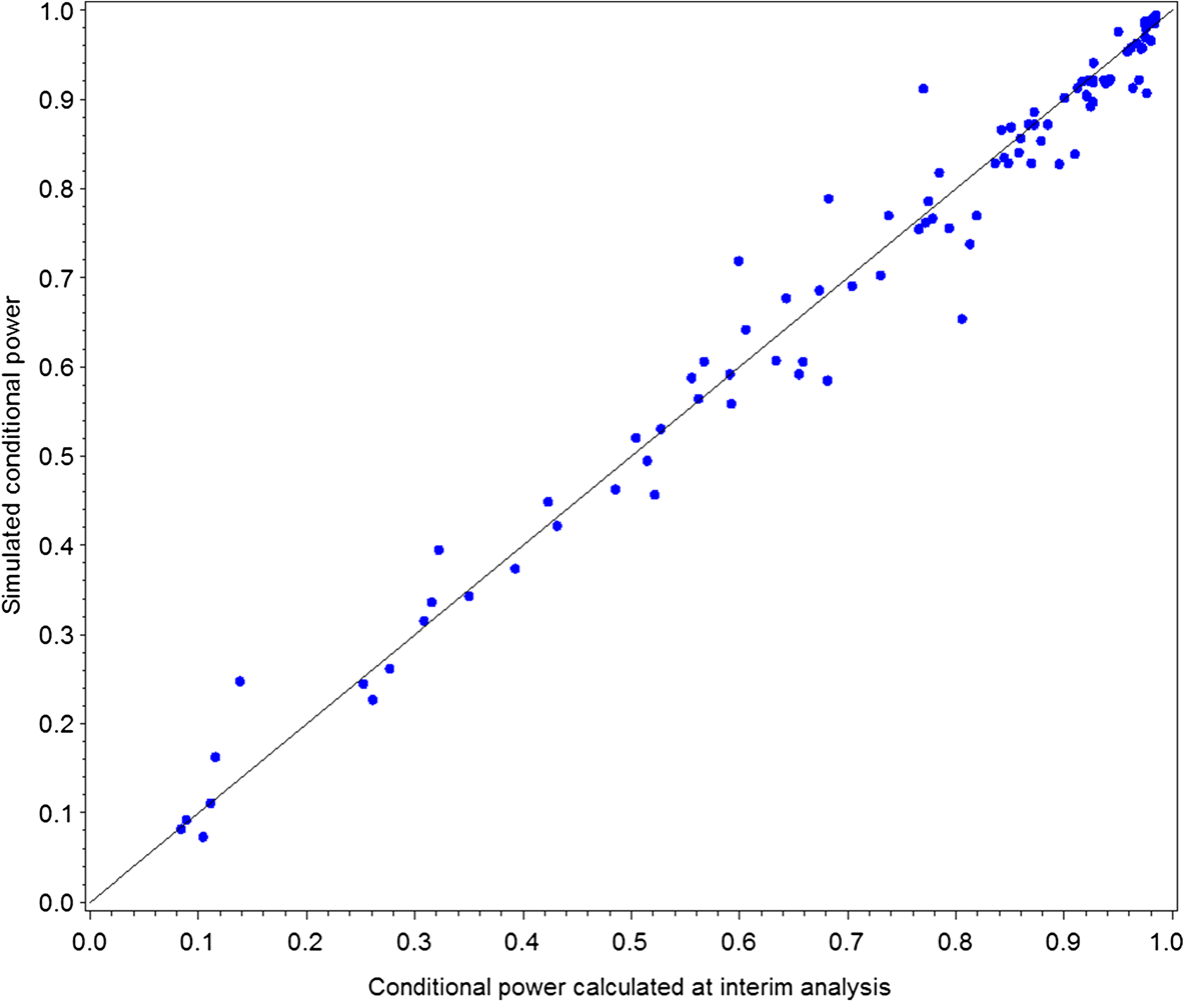
**Results of the conditional power validation displaying a plot of the empirical conditional power (y-axis) and calculated conditional power calculated at the interim analysis (x-axis).** The solid line represents the y = x line.

To ensure that the modification the conditional power formula (formula 9) was appropriate, we performed a validation study using simulations. For each combination of prevalence of the prognostic factor and magnitude of the interaction ran 10 trials to obtain 10 interim test statistics for each combination of parameters. At the interim analysis we calculated the conditional power based on the hypothesized values of *θ*, *σ*, and *p*_*1*_. For each trial, the second half of the trial was simulated 5,000 times to obtain the empirical conditional power. Since there were 10 different combinations of prevalence of the prognostic factor and magnitude of the interaction effect and 10 trials for each combination, the plot generated 100 points. We generated a scatter plot of the empirical conditional power based on 5,000 replicates against the calculated conditional power (Figure 1). Values that line up along the y = x line demonstrate that formula provided an accurate estimation of the conditional power.

**Figure 2:**
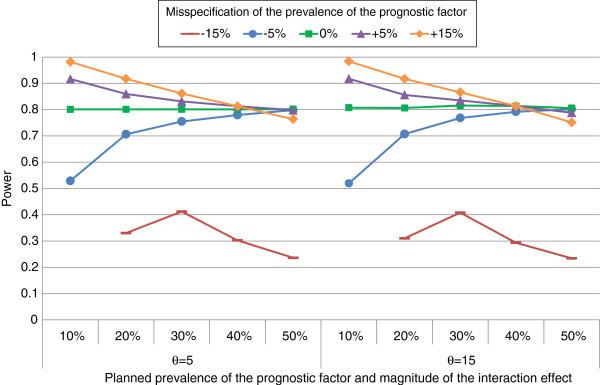
Power using traditional study design by magnitude of the interaction effect, planned prevalence of the prognostic factor, and misspecification of the prevalence of the prognostic factor.

### Simulation study details

Five thousand replications were performed for each combination of the interaction effect and proportion at level *k*_*1*_. We first evaluated the empirical power for detecting the interaction effect without accounting for misspecification of the distribution of the prognostic factor. We varied the misspecification of the prognostic factor at −15%, -5%, 0%, +5%, and +15%. For the quota sampling method we did not vary the misspecification of the distribution of the prognostic factor because the definition of the method does not allow for misspecifications. While we did not expect the quota sampling method to have power or type I error estimates that differ from the traditional one-stage sampling design under no misspecification, we conducted the simulation study for this study design method to confirm there was no impact on power and type I error. For the modified quota sampling method and sample size re-estimation using conditional power we used the same misspecifications as described above.

We calculated the overall empirical power for the interaction effect for all three methods. This was defined as the percentage of statistically significant interaction effects across the 5,000 replicates. Empirical type I error was calculated in a similar fashion for these three methods, but the interaction effect was assumed to be zero and the sample size we used was the sample sizes for the planned interaction effects of 15 and 5. For the sample size re-estimation method we also calculated the empirical conditional power. This was defined as the number of statistically significant interaction effects detected at the 0.05 level among trials that re-estimated the sample size. Because the sample size could change, we also calculated the mean and median final sample size for the entire procedure.

The margin of error for empirical power and type I error was calculated using the half width of the 99% confidence interval based on a binomial distribution with a sample size of 5,000. Since trials were planned with 1– *β* = 0.80 *α* = 0.05 this led to margins of error equal of 0.015 and 0.008 when assessing empirical power and type I error respectively.

## Results

### Effect of misspecifying the distribution of the prognostic factor on power for the interaction test

Power curves when using the traditional study design are shown in Figure 2. There was a small difference in power when comparing the magnitude of the interaction effect and holding the planned prevalence of the prognostic factor and the misspecification of the prognostic factor equal. This was due to rounding up of the final sample size and the choice of using the t-critical values instead of the z-critical values, which were larger when the magnitude of the interaction was larger. In short, if the actual prevalence is closer to 50%, the power is higher than planned, and if the actual prevalence is farther away from 50%, the power is lower than planned, see also Figure 2 and equation 4.

### Performance of the quota sampling procedure

The quota sampling procedure performed well in terms of empirical power (Table 1) and type I error (Table 2) as a strategy to account for misspecifying the distribution of the prognostic factor. The empirical power was reached or exceeded the target power of 80% for all combinations of *θ* and distributions of the prognostic factor. The type I error was near the target type I error of 5% for all combinations of sample size and distributions of the prognostic factor.

**Table 1:** Empirical power for all three methods when there was no misspecification of the distribution of the prognostic factor

***θ***	**Planned distribution of the prognostic factor**	**Planned *n***_***t***_	**Quota sampling**	**Modified quota sampling**	**Sample size re-estimation using conditional power**
5	10%	1,418	0.8088	0.8054	0.8836
	20%	798	0.8152	0.8082	0.8916
	30%	608	0.8178	0.8014	0.8870
	40%	532	0.8116	0.8036	0.8866
	50%	512	0.8156	0.8098	0.8974
15	10%	178	0.8556	0.8204	0.8890
	20%	100	0.8490	0.8128	0.8930
	30%	78	0.8442	0.8316	0.9012
	40%	68	0.8556	0.8322	0.9038
	50%	64	0.8412	0.8256	0.9082

The margin of error based on the 99% confidence interval is 0.015.

**Table 2:** Empirical type I error for all three methods when there was no misspecification of the distribution of the prognostic factor

***θ***	**Planned distribution of the prognostic factor**	**Planned *n***_***t***_	**Quota sampling**	**Modified quota sampling**	**Sample size re-estimation using conditional power**
0	10%	1,418	0.0496	0.0534	0.0210
	20%	798	0.0484	0.0522	0.0260
	30%	608	0.0494	0.0464	0.0286
	40%	532	0.0510	0.0514	0.0302
	50%	512	0.0532	0.0540	0.0248
0	10%	178	0.0494	0.0512	0.0248
	20%	100	0.0488	0.0516	0.0274
	30%	78	0.0536	0.0524	0.0224
	40%	68	0.0472	0.0496	0.0302
	50%	64	0.0482	0.0482	0.0328

The margin of error based on the 99% confidence interval is 0.008.

### Performance of the modified quota sampling procedure

Under no misspecification of the distribution of the prognostic factor, the modified quota sampling procedure performed well with empirical power greater than or equal to 80% across all situations (Table 1). The type I error rate was also near 5% for all combinations under no misspecification (Table 2).

Under negative misspecifications of the distribution of the prognostic factor, empirical power was improved in comparison to doing nothing but 80% power was not achieved in all cases (Table 3). The ability of the procedure to attain 80% power under misspecifications of the prognostic factor was dependent on the percentage of trials that switched to quota sampling after 50% enrollment. The likelihood of switching to quota sampling was related to the magnitude of the interaction effect, the planned distribution of the prognostic factor, and the magnitude of the negative misspecification. When the magnitude of the interaction effect was five and the misspecification of the distribution of the prognostic factor was −15%, greater than 99.8% of the trials switched to the quota sampling method and the procedure attained 80% power. However, when the magnitude of the interaction effect was 15, and the misspecification of the distribution of the prognostic factor was −5%, the modified quota sampling approach only attained 80% power when the planned distribution of the prognostic factor was 40% or 50% (Table 3).

**Table 3:** Empirical power and percentage of trials switching to the quota sampling scheme for the modified quota sampling method

***θ***	**Planned distribution of the prognostic factor**	**Planned *n***_***t***_	**Empirical power**	**Percent of trials switching to quota sampling**
*Misspecification of the prognostic factor: -5%*				
5	10%	1,418	0.8012	99.98%
	20%	798	0.7736	74.08%
	30%	608	0.7802	47.52%
	40%	532	0.7932	36.64%
	50%	512	0.8060	35.50%
15	10%	178	0.6636	33.04%
	20%	100	0.7400	11.06%
	30%	78	0.7940	11.84%
	40%	68	0.8164	11.90%
	50%	64	0.8122	8.48%
*Misspecification of the prognostic factor: -15%*				
5	10%	1,418	--	--
	20%	798	0.8022	100.00%
	30%	608	0.8024	100.00%
	40%	532	0.8058	99.94%
	50%	512	0.8090	99.82%
15	10%	178	--	--
	20%	100	0.7788	89.06%
	30%	78	0.7834	63.64%
	40%	68	0.7904	49.70%
	50%	64	0.8038	40.00%
*Misspecification of the prognostic factor: +5%*				
5	10%	1,418	0.8000	98.28%
	20%	798	0.8090	67.72%
	30%	608	0.8278	49.32%
	40%	532	0.8092	37.08%
	50%	512	0.7984	36.30%
15	10%	178	0.8880	35.14%
	20%	100	0.8656	16.62%
	30%	78	0.8542	10.94%
	40%	68	0.8350	7.86%
	50%	64	0.8198	8.60%
*Misspecification of the prognostic factor: +15%*				
5	10%	1,418	0.8738	100.00%
	20%	798	0.8010	100.00%
	30%	608	0.8092	99.98%
	40%	532	0.8088	99.80%
	50%	512	0.8064	99.86%
15	10%	178	0.8942	97.82%
	20%	100	0.8504	72.44%
	30%	78	0.8572	50.72%
	40%	68	0.8466	38.48%
	50%	64	0.8174	40.88%

The margin of error based on the 99% confidence interval is 0.015.

For positive misspecifications of the distribution of the prognostic factor, the modified quota sampling procedure attained 80% for all combinations of the magnitude of the interaction effect and planned distribution of the prognostic factor (Table 3).

Type I error was maintained at 5% or within the margin of error for all combinations of sample size, planned distribution of the prognostic factor, and misspecification of the distribution of the prognostic factor (Table 4).

**Table 4:** Empirical type I error and percentage of trials switching to the quota sampling scheme for the modified quota sampling method

***θ***	**Planned distribution of the prognostic factor**	**Planned *n***_***t***_	**Empirical type I error**	**Percent of trials switching to quota sampling**
*Misspecification of the prognostic factor: -5%*				
0	10%	1,418	0.0528	99.90%
	20%	798	0.0506	74.26%
	30%	608	0.0498	47.34%
	40%	532	0.0480	36.66%
	50%	512	0.0492	36.06%
0	10%	178	0.0566	34.74%
	20%	100	0.0496	12.12%
	30%	78	0.0528	11.24%
	40%	68	0.0452	11.30%
	50%	64	0.0522	8.80%
*Misspecification of the prognostic factor: -15%*				
0	10%	1,418	--	--
	20%	798	0.0500	100.00%
	30%	608	0.0496	100.00%
	40%	532	0.0518	99.92%
	50%	512	0.0568	99.76%
0	10%	178	--	--
	20%	100	0.0578	89.60%
	30%	78	0.0522	62.82%
	40%	68	0.0542	50.56%
	50%	64	0.0478	41.20%
*Misspecification of the prognostic factor: +5%*				
0	10%	1,418	0.0568	98.68%
	20%	798	0.0490	68.74%
	30%	608	0.0490	48.10%
	40%	532	0.0508	36.48%
	50%	512	0.0508	36.64%
0	10%	178	0.0504	34.46%
	20%	100	0.0534	16.32%
	30%	78	0.0496	10.90%
	40%	68	0.0450	8.50%
	50%	64	0.0502	8.94%
*Misspecification of the prognostic factor: +15%*				
0	10%	1,418	0.0462	100.00%
	20%	798	0.0490	100.00%
	30%	608	0.0484	100.00%
	40%	532	0.0516	99.76%
	50%	512	0.0522	99.86%
0	10%	178	0.0452	97.48%
	20%	100	0.0444	70.72%
	30%	78	0.0526	51.00%
	40%	68	0.0524	38.60%
	50%	64	0.0528	40.72%

The margin of error based on the 99% confidence interval is 0.008.

### Validating the conditional power formula

Figure 1 shows the validation results of the conditional power formula used in this paper. The points line up along the y = x line, which implies that the formula we used to calculate the conditional power was similar to the empirical conditional power. These results give us confidence that the sample size re-estimation presented in the next section performed as expected.

### Performance of the sample size re-estimation using conditional power procedure

Under no misspecification of the distribution of the prognostic factor, using the sample size re-estimation procedure resulted in an increase in overall power due to the requirement of conditional power of 80% at the interim analysis. Across different combinations *θ* and the planned distribution of the prognostic factor, the empirical power ranged between 88% and 91% (Table 1). Despite the increase in power, type I error was maintained at 5% or less for all of the simulations under no misspecification of the distribution of the prognostic factor (Table 2).

When we assumed there was a misspecification of −5% for the distribution of the prognostic factor, the empirical power was greater than 80% except when the planned distribution of the prognostic factor was 10%. In this case the empirical power was 72%-73%, which was an improvement compared to using the traditional one-stage design (Figure 2). For a misspecification of the distribution of the prognostic factor of −15% and planned prognostic factor distribution of 20%, the empirical power was also less than 80% (empirical power of 56%-60%), but was higher than using the traditional one-stage design (Figure 2). The inability to attain 80% power in these situations was directly related to the fact that more trials were stopped for futility at the interim analysis. In the situations where the empirical power failed to attain 80% power the percentage of trials stopping for futility ranged between 15% and 26% (Table 5).

**Table 5:** Empirical power and the percentage of trials stopping for futility and efficacy for sample size re-estimation using conditional power

***θ***	**Planned distribution of the prognostic factor**	**Planned *n***_***t***_	**Empirical power**	**Percent stopping for futility**	**Percent stopping for efficacy**
*Misspecification of the prognostic factor: -5%*					
5	10%	1,418	0.7324	16.58%	5.76%
	20%	798	0.8410	10.34%	11.76%
	30%	608	0.8616	9.02%	13.72%
	40%	532	0.8754	8.24%	15.42%
	50%	512	0.8868	7.64%	16.22%
15	10%	178	0.7246	17.14%	7.90%
	20%	100	0.8232	12.26%	11.82%
	30%	78	0.8736	8.94%	14.76%
	40%	68	0.8936	7.72%	15.80%
	50%	64	0.9010	7.10%	16.38%
*Misspecification of the prognostic factor: -15%*					
5	10%	1,418	--	--	--
	20%	798	0.5614	26.06%	3.5%
	30%	608	0.7536	15.46%	7.3%
	40%	532	0.8304	10.72%	10.80%
	50%	512	0.8750	8.34%	14.32%
15	10%	178	--	--	--
	20%	100	0.6026	22.68%	4.96%
	30%	78	0.7634	15.76%	8.50%
	40%	68	0.8412	10.78%	11.86%
	50%	64	0.8734	9.32%	14.76%
*Misspecification of the prognostic factor: +5%*					
5	10%	1,418	0.9528	3.10%	27.44%
	20%	798	0.9194	5.58%	20.94%
	30%	608	0.9064	6.26%	18.36%
	40%	532	0.8898	7.18%	17.16%
	50%	512	0.8868	7.52%	16.02%
15	10%	178	0.9476	3.94%	29.36%
	20%	100	0.9194	5.70%	21.78%
	30%	78	0.9204	5.94%	20.38%
	40%	68	0.9146	6.36%	18.28%
	50%	64	0.9056	6.96%	15.88%
*Misspecification of the prognostic factor: +15%*					
5	10%	1,418	0.9868	0.94%	45.24%
	20%	798	0.9494	3.38%	27.26%
	30%	608	0.9186	5.34%	20.84%
	40%	532	0.8990	6.98%	17.12%
	50%	512	0.8822	8.00%	14.30%
15	10%	178	0.9902	0.78%	49.94%
	20%	100	0.9584	3.18%	29.86%
	30%	78	0.9412	4.32%	22.32%
	40%	68	0.9108	6.56%	17.12%
	50%	64	0.8816	8.76%	13.84%

The margin of error based on the 99% confidence interval is 0.015.

The empirical type I error was below 5%, suggesting room for power to gain by changing the critical values of *c*_*1*_ and *c*_*2*_ for all combinations of *θ*, planned distribution of the prognostic factor, and misspecification of the distribution of the prognostic factor. Under the null hypothesis, the percentage of trials stopping for futility ranged between 42% and 45%, while the percentage of trials stopping for efficacy was at most 0.6% (Table 6).

**Table 6:** Empirical type I error and the percentage of trials stopping for futility and efficacy for sample size re-estimation using conditional power

***θ***	**Planned distribution of the prognostic factor**	**Planned *n***_***t***_	**Empirical type I error**	**Percent stopping for futility**	**Percent stopping for efficacy**
*Misspecification of the prognostic factor: -5%*					
0	10%	1,418	0.0278	43.48%	0.24%
	20%	798	0.0264	44.54%	0.26%
	30%	608	0.0262	42.66%	0.20%
	40%	532	0.0280	44.16%	0.24%
	50%	512	0.0246	42.88%	0.30%
0	10%	178	0.0304	42.94%	0.40%
	20%	100	0.0276	42.44%	0.28%
	30%	78	0.0252	43.16%	0.54%
	40%	68	0.0230	43.08%	0.44%
	50%	64	0.0298	42.00%	0.56%
*Misspecification of the prognostic factor: -15%*					
0	10%	1,418	--	--	--
	20%	798	0.0254	43.62%	0.24%
	30%	608	0.0278	42.94%	0.30%
	40%	532	0.0282	44.00%	0.36%
	50%	512	0.0248	42.62%	0.32%
0	10%	178	--	--	--
	20%	100	0.0326	44.24%	0.50%
	30%	78	0.0282	42.52%	0.44%
	40%	68	0.0286	42.62%	0.42%
	50%	64	0.0296	43.26%	0.50%
*Misspecification of the prognostic factor: +5%*					
0	10%	1,418	0.0314	41.46%	0.36%
	20%	798	0.0284	42.94%	0.22%
	30%	608	0.0296	44.76%	0.44%
	40%	532	0.0312	43.82%	0.32%
	50%	512	0.0290	44.16%	0.36%
0	10%	178	0.0284	43.64%	0.30%
	20%	100	0.0274	43.30%	0.38%
	30%	78	0.0296	43.66%	0.44%
	40%	68	0.0280	44.24%	0.48%
	50%	64	0.0290	43.82%	0.46%
*Misspecification of the prognostic factor: +15%*					
0	10%	1,418	0.0244	43.32%	0.28%
	20%	798	0.0274	44.44%	0.34%
	30%	608	0.0232	43.98%	0.20%
	40%	532	0.0290	43.18%	0.32%
	50%	512	0.0284	44.22%	0.32%
0	10%	178	0.0282	43.36%	0.38%
	20%	100	0.0282	42.50%	0.36%
	30%	78	0.0266	43.92%	0.34%
	40%	68	0.0266	44.30%	0.46%
	50%	64	0.0248	42.48%	0.34%

The margin of error based on the 99% confidence interval is 0.008.

Conditional properties are displayed in Table 7. In almost all cases the empirical conditional power greater than 80%. The two situations in which the empirical conditional power was less than 80% was when there was a negative misspecification of −15% of the distribution of the prognostic factor coupled with an initial planned distribution of the prognostic factor of 20%. Here the empirical conditional power was 76% for *θ* equal to 5 and 15. The mean total sample size was always greater than the original planned sample size. Some of these mean total sample sizes were more than double the final sample size. However, the median sample size was equal to or very close to the original total sample size in all cases (Table 7).

**Table 7:** Percentage of trials re-estimating the sample size, conditional power among trials that re-estimated the sample size and overall mean and median sample size for sample size re-estimation using conditional power

***θ***	**Planned distribution of the prognostic factor**	**Planned *n***_***t***_	**Percent re-estimating sample size**	**Empirical conditional power**	**Mean sample size**	**Median sample size**
*Misspecification of the prognostic factor: 0%*						
5	10%	1,418	37.44%	0.9471	2,500	1,418
	20%	798	37.30%	0.9566	1,470	798
	30%	608	37.74%	0.9671	1,143	608
	40%	532	38.00%	0.9689	984	532
	50%	512	39.44%	0.9660	989	512
15	10%	178	37.44%	0.9701	327	178
	20%	100	40.48%	0.9674	202	100
	30%	78	39.28%	0.9695	150	78
	40%	68	42.02%	0.9719	155	68
	50%	64	43.58%	0.9748	154	64
*Misspecification of the prognostic factor: -5%*						
5	10%	1,418	49.70%	0.8881	3,568	1,418
	20%	798	43.12%	0.9429	1,696	798
	30%	608	40.98%	0.9458	1,231	608
	40%	532	38.56%	0.9570	1,062	532
	50%	512	38.82%	0.9629	987	512
15	10%	178	45.00%	0.8738	402	178
	20%	100	42.54%	0.9342	219	100
	30%	78	43.56%	0.9660	171	78
	40%	68	43.38%	0.9779	153	68
	50%	64	44.40%	0.9770	155	64
*Misspecification of the prognostic factor: -15%*						
5	10%	1,418	--	--	--	--
	20%	798	51.98%	0.7618	2,425	865
	30%	608	47.34%	0.8952	1,439	608
	40%	532	44.96%	0.9346	1,155	532
	50%	512	41.42%	0.9546	1,028	512
15	10%	178	--	--	--	--
	20%	100	48.70%	0.7573	267	100
	30%	78	46.80%	0.9167	190	78
	40%	68	47.38%	0.9565	161	68
	50%	64	44.50%	0.9685	148	64
*Misspecification of the prognostic factor: +5%*						
5	10%	1,418	25.94%	0.9861	1,984	1,418
	20%	798	33.26%	0.9747	1,310	798
	30%	608	35.76%	0.9676	1,096	608
	40%	532	37.68%	0.9618	975	532
	50%	512	39.24%	0.9623	969	512
15	10%	178	28.32%	0.9845	263	178
	20%	100	35.36%	0.9762	177	100
	30%	78	37.42%	0.9840	153	78
	40%	68	43.32%	0.9797	150	68
	50%	64	45.72%	0.9790	157	64
*Misspecification of the prognostic factor: +15%*						
5	10%	1,418	15.04%	0.9960	1,472	1,418
	20%	798	27.34%	0.9824	1,159	798
	30%	608	34.22%	0.9673	1,007	608
	40%	532	38.58%	0.9725	1,026	532
	50%	512	41.56%	0.9644	1,045	512
15	10%	178	14.40%	0.9944	190	89
	20%	100	30.38%	0.9888	159	100
	30%	78	37.62%	0.9856	149	78
	40%	68	43.78%	0.9753	151	68
	50%	64	48.00%	0.9708	168	64

## Discussion

We evaluated the impact of misspecifying the distribution of a prognostic factor on the power and sample size for interaction effects in an RCT setting. We showed that negative misspecification of the distribution of the prognostic factor resulted in a loss of power and a need for an increased sample size, because the planned distribution of the prognostic factor moved further away from a balanced design.

We evaluated three methods for handling misspecifying the distribution of the prognostic factor when investigating interaction effects in an RCT setting. The first two methods dealt with how the subjects would be sampled. The quota sampling method removed any variability in the prognostic factor and by definition misspecification of the distribution of the prognostic factor was not possible. For example if a trial was set to enroll 200 subjects with 30% in the *k*_*1*_ level of the prognostic factor then enrollment would be capped at 60 subjects in the *k*_*1*_ level and 140 in the *k*_*2*_ level. This method did a good job of maintaining the power at 80% and controlling the type I error at 5%. The modified quota sampling approach did not perform as well in all situations. In summary, this method enrolled subjects randomly for the first half the trial. The sampling method would switch to the quota sampling approach if the distribution of the prognostic factor differed significantly from what was planned. Power was maintained at 80% when the percentage of trials switching to the quota sampling approach was large. However, when the percentage switching was small and there was a negative misspecification of the distribution of the prognostic factor, the power was compromised, but rarely substantially.

The last method used conditional power at an interim analysis (after 50% enrollment) to re-estimate the sample size. We adapted the method used by Denne [10]. This method resulted in more optimal overall power and type I error estimates than the modified quota sampling procedure. The main reason this method could not maintain empirical power at 80% under a negative misspecification is that too many trials stopped for futility before the sample size could be re-estimated and all of the type II error was used up at the interim analysis. This result does not diminish the value of the sample size re-estimation procedure since misspecification of other trial parameters that diminish power would have a similar effect.

The findings from our study detail methods for handling the misspecification of the distribution of a prognostic factor when detecting an interaction effect in an RCT setting. An advantage of the quota sampling approach over using the conditional power formula is that the final sample size does not need to be changed and an interim analysis that uses some of the alpha level does not need to be undertaken. However, the quota sampling approach is sensitive to distortions in misspecifying the distribution of the prognostic factor in terms of trial duration as it may take longer to recruit the necessary patients from the appropriate level of the prognostic factor. Using sample size re-estimation does not have this issue, but it is sensitive to the values used in the conditional power formula. In particular, the mean sample size tended to be larger the original planned sample size in situations where the misspecification was leading to a more balanced design. In theory, this should increase the power and reduce the sample size needed. However, in some of the simulations in which the conditional power was low, but did not reach the futility stopping rule, the new sample size estimated was very large, resulting in an outlier. These outliers inflated the final mean sample size. To overcome this, we also reported the median final sample size of the simulations, which was less than or equal to the originally planned sample size.

As with any study that uses simulations, there are several limitations to our study. One limitation is that not all interaction effects were explored and we only study a 2 by 2 interaction effect. However summarizing the interaction effect with one contrast would not be feasible if three or more treatments or levels of the prognostic factor were explored. Future work could explore looking at these interaction effects. We also did not examine the impact of informative cross-over. Many times subjects randomized to one arm (ex. non-surgical therapy) in a study will cross-over to another arm (ex. surgical therapy). The impact of differing cross-over rates should be explored.

Another limitation is that we did not look at the impact of unequal variances. This should be the goal of future work as levels of a prognostic factor can impact variability in the outcome.

Lastly, we only studied the impact of one sample size re-estimation procedure as described by Denne in 2001 [10]. However, there are many different methods of re-estimating the sample size that could be studied [12]. However, the method described by Denne still is a valid and acceptable method according to the FDA guidelines on Adaptive Design Clinical Trials for Drugs and Biologics [13].

## Conclusions

We examined three methods for dealing with the misspecification of the distribution of the prognostic factor when determining the treatment by prognostic factor interaction effects in an RCT setting. Sample size re-estimation using conditional power was able to improve the power when there was a negative misspecification of the distribution of the prognostic factor while maintaining appropriate type I error. As more studies seek to explore interaction effects as their primary outcome in RCTs, these methods will be useful for clinicians planning their studies. Further research should look at the impact of cross-over between treatment groups.

## Competing interests

The authors declare that they have no competing interests.

## Authors’ contributions

WMR designed the simulation study, interpreted the data, and drafted the manuscript. MPL interpreted the data and critically revised the manuscript. DRG interpreted the data and critically revised the manuscript. EL interpreted the data and critically revised the manuscript. All authors read and approved the final manuscript.

## Grant support

This research was supported in part by the National Institutes of Health, National Institute of Arthritis and Musculoskeletal and Skin Diseases grants T32 AR055885 and K24 AR057827.

## Pre-publication history

The pre-publication history for this paper can be accessed here:

http://www.biomedcentral.com/1471-2288/13/21/prepub
